# Descemet’s membrane endothelial keratoplasty for acute corneal hydrops: a case report

**DOI:** 10.5935/0004-2749.20200085

**Published:** 2024-02-11

**Authors:** Lucio V. L. Maranhão, Natália Regnis L. Ramalho, Wanessa M. P. Pinto, Paulo Elias Correa Dantas, Camila V. Ventura

**Affiliations:** 1 Department of Ophthalmology, Fundação Altino Ventura, Recife, PE, Brazil; 2 Department of Ophthalmology, Hospital de Olhos de Pernambuco, Recife, PE, Brazil; 3 Department of Ophthalmology and Visual Sciences, Escola Paulista de Medicina, Universidade Federal de São Paulo, São Paulo, SP, Brazil

**Keywords:** Keratoconus/surgery, Descemet stripping endothelial keratoplasty, Slit-lamp microscopy, Ceratocone/cirurgia, Ceratoplastia endotelial com remoção da lâmina limitante posterior Microscopia com lâmpada de fenda

## Abstract

Here, we describe the result of a Descemet’s membrane endothelial keratoplasty for acute corneal hydrops in a 45-year-old female with keratoconus, who presented with severe visual loss in her OS. The patient’s best-corrected visual acuity was 20/80 in the right eye and hand motion in the OS. Slit-lamp examination revealed an extensive tear of the Descemet’s membrane and stromal corneal edema in the OS. We opted for Descemet membrane endothelial keratoplasty. Twelve months postoperatively, the patient had a best-corrected visual acuity of 20/50 in the OS.

## Abstract (pt)

Trata-se de uma paciente do sexo feminino, de 45 anos, portadora de ceratocone, submetida a uma ceratoplastia endotelial com membrana Descemet após apresentar um quadro de perda de visão severa devido a uma hidrópsia corneana aguda no olho esquerdo. Inicialmente, a acuidade visual corrigida da paciente era de 20/80 no olho direito e de movimento de mãos no olho esquerdo. Após exame de biomicroscopia que detectou uma extensa rotura da membrana de Descemet e edema estromal, optamos por tratar esse caso com o ceratoplastia endotelial com membrana Descemet. Doze meses após o procedimento cirúrgico, percebeu-se uma melhora do edema corneano, não havia sinais de rejeição do botão óptico e a acuidade visual corrigida da paciente era de 20/50 no olho afetado.

## INTRODUCTION

Keratoconus is an ectasic corneal disorder related to progressive thinning of the stroma, which impairs vision because of irregular astigmatism and corneal scarring. The etiology is not clear, but its development involves pro-inflammatory factors and complex interactions between genetic and environmental factors^(1^,^2)^.

Acute corneal hydrops is a complication of advanced corneal ectasia and is characterized by localized corneal edema secondary to persistent stress and spontaneous tear of Descemet’s membrane (DM). Once torn, the DM might retract and curl anteriorly, favoring aqueous entry into the corneal stroma and causing acute visual loss and sudden ocular pain^(3)^. Despite being unusual, acute corneal hydrops is considered a self-healing disorder that usually resolves in 3 months and leaves a scar. Depending on its size, opacity, and contour, the scar might lead to contact lens intolerance and require corneal transplantation to restore visual function^(3-5)^.

During the acute phase of corneal hydrops, conventional therapy (i.e., hypertonic saline eye drops and to pical steroids) to reduce pain and inflammation is considered the first-line treatment. A small number of patients with large clefts and severe edema may persist for long periods and might require surgical intervention because of residual corneal opacity^(6)^. Surgical procedures to treat acute corneal hydrops have the objective of providing faster recovery and minimizing complications. However, they have limitations, and often, penetrating keratoplasty (PK) is required to restore corneal transparency and visual function^(7-10)^.

In this report, we described an acute case of corneal hydrops treated with the DM endothelial keratoplasty (DMEK), which resolved corneal hydrops and resulting in visual recovery.

## CASE REPORT

A 45-year-old woman with a history of keratoconus and a previous best-corrected visual acuity (BCVA) of 20/20 in both eyes (oculus uterque [OU]) and well adapted to rigid contact lenses presented with progressive visual loss and intolerance to her contact lenses in the left eye (oculus sinister [OS]) over a period of 24 h. She was referred to the Department of Cornea at the Altino Ventura Foundation, Recife, Brazil, after an unsuccessful response to a 30-day treatment with hypertonic and anti-inflammatory drops. She denied previous ocular procedures in the OU.

**Figure 1:**
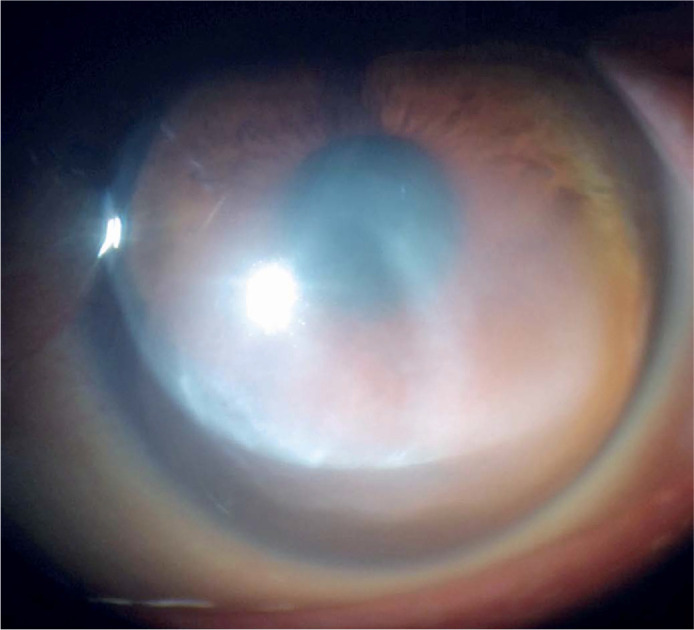
Slit-lamp image prior to surgery, showing a large DM tear and

On examination, her BCVA was 20/80 in the right eye (oculus dextrus [OD]) and hand motion in the OS. Slit-lamp examination revealed corneal ectasia in the OU and a large inferior 3 mm DM tear associated with stromal corneal edema (3+/4+) affecting the OS visual axis (Figure 1). Optical pachymetry at presentation showed that the central corneal thickness (CCT) was 426 µm in the OD and 659 µm in the OS.

A month of conventional treatment with topical 5% sodium chloride *hypertonic* and prednisolone eye drops did not achieve visual improvement, so different surgical options were discussed with the patient and DMEK was selected.

The procedure was performed by an experienced cornea surgeon (L.V.L.M.). No dehydration technique was used prior to surgery. Under topical anesthesia, a clear 3 mm corneal incision and two paracenteses were initially performed. The anterior chamber (AC) was filled with cohesive viscoelastic; a reverse Sinskey hook was used to perform an 8.5 mm circular descemetorhexis. The descemetorhexis was difficult to perform because of corneal edema but was about the same size as the corneal edema. The donor DMEK graft was stripped from an 8 mm trephined cornea stained with trypan blue (Ophthalmos SA, São Paulo, Brazil) and loaded into a Geuder AG injector.

**Figure 2:**
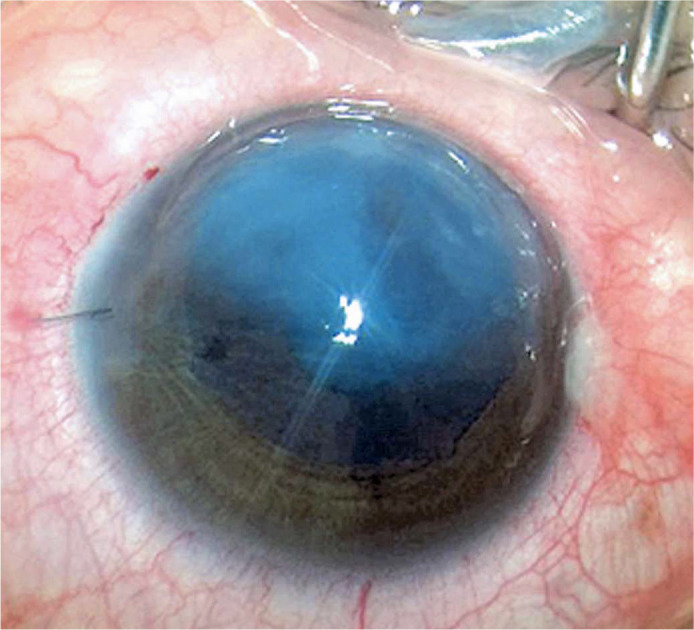
Immediate postoperative image showing a centralized graft hydrops in the OS. DM, Descemet’s membrane; OS, oculus sinister (left eye). covering the hydrops area.

Inferior iridectomy was performed, the viscoelastic was completely aspirated from the AC, and the DMEK donor graft was inserted with no difficulty. Then, the DMEK donor graft was centralized to cover the entire extension of the DM cleft (Figure 2). The AC was completely filled with 11% perfluoropropane gas (C_3_F_8_) and a 10-0 nylon suture was used to close the main corneal incision.

**Figure 3:**
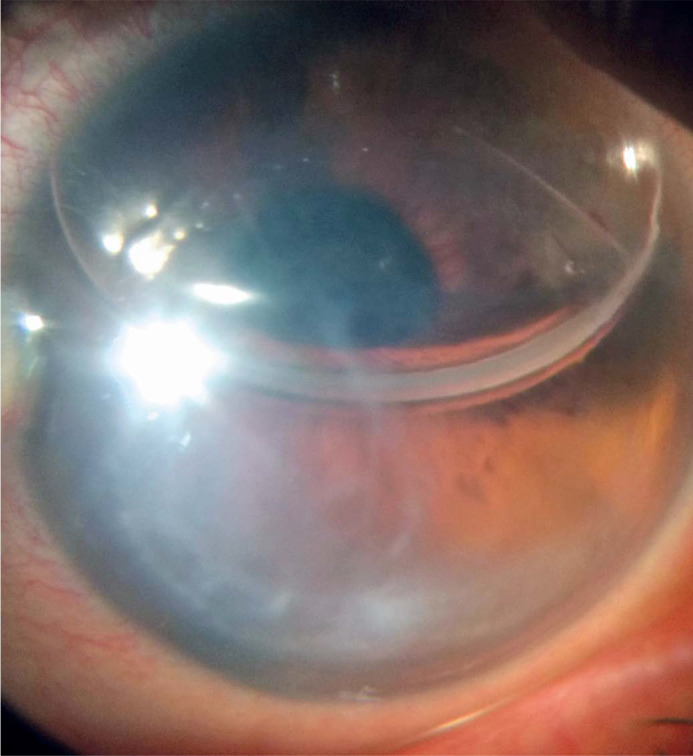
Corneal edema improvement and 50% of the AC filled with 11% C_3_F_8_ at postoperative day 7. AC, anterior chamber.

**Figure 4:**
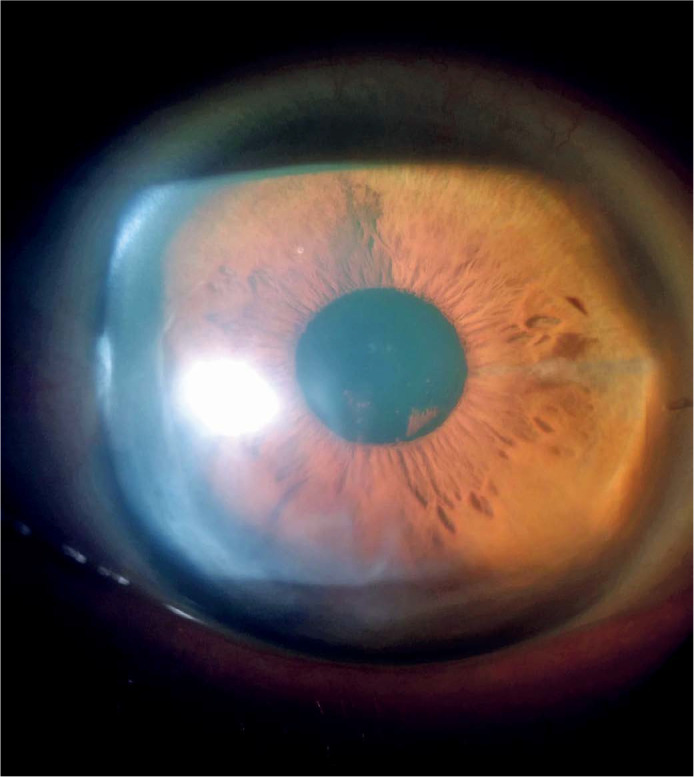
Slit-lamp image of the OS, showing corneal transparency 3 months after DMEK. OS, oculus sinister (left eye); DMEK, Descemet’s membrane endothelial keratoplasty.

**Figure 5:**
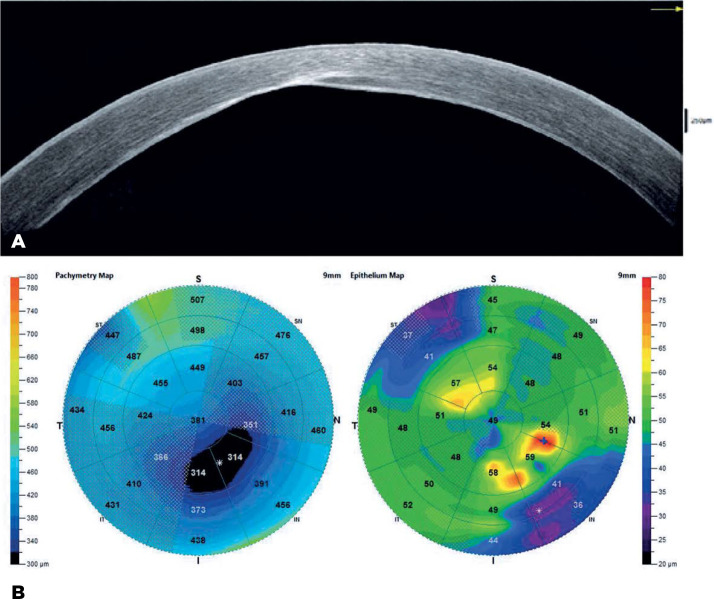
A) OCT image of the OS, showing an attached graft over the affected area. B) OCT pachymetry map showing diffuse reduction of corneal edema in the OS (576 _µ_m).

On postoperative day 7, we noted improvement in corneal edema and cloudiness, the DMEK graft was still attached, and the gas-fill was 50% in the AC (Figure 3). Two months postoperatively, the patient’s BCVA in the OS with a scleral contact lens was 20/50. Slit-lamp examination and anterior segment ocular coherence tomography (AS-OCT) imaging showed an attached DMEK graft (Figures 4 and 5 A,B). The CCT variation in the OS at 2 months postoperatively showed a decrease in corneal edema in the hydrops area (576 µm).

OCT= ocular coherence tomography; OS= oculus sinister (left eye).

On the 12^th^ month follow-up, the patient maintained both the integrity of the DMEK and the BCVA.

## DISCUSSION

According to Pena et al., the overall rate of acute hydrops in keratoconus cases is 5.8%^(11)^. Although it usually resolves spontaneously, acute corneal hydrops commonly leaves a corneal scar that compromises visual acuity^(3)^. These cases progress to corneal scaring and difficulty in adapting to contact lenses, and PK is commonly indicated to improve vision. However, PK is an invasive procedure and has the risk of rejection and potential neovascularization^(6)^. Therefore, to provide faster recovery, alternative surgical interventions have been experimented with in the past years.

Gas injection of 11% C_3_F_8_, whether associated or not associated with compressive corneal sutures, can be used but even that has limitations depending on the DM tear location and extension, as well as intrastromal involvement. In addition, compressive corneal sutures do not provide corneal transparency^(7-9)^.

Palioura et al. described a case of an acute hydrops patient with keratoglobus treated with a large Descemet’s stripping automated endothelial keratoplasty (DSAEK) graft^(12)^. The initial suture-affixed DSAEK procedure could not permanently resolve the DM tear, and a second suture-affixed DSAEK procedure was required. Despite being technically easy to perform, even in cases with bad visualization of the AC, DSAEK may result in a worse visual prognosis because of the graft thickness and sutures used for graft fixation. In our case, the graft was thin, did not require graft sutures, adhered in the first attempt, covered the entire affected area, and restored the cornea’s transparency and the patient’s vision.

Recently, Elmer Y. Tu^(13)^ reported a case of chronic corneal hydrops, of 7 months’ duration, in which DMEK was performed and at an 18-month follow-up showed hydrops resolution, a stable graft, and clear cornea^(13)^.

Despite the technical challenges in DMEK, especially when visualization is compromised, it may be a reasonable alternative for acute corneal hydrops management because it preserves corneal integrity, favors corneal transparency, and provides faster anatomical and visual recovery.

## References

[1] Martínez-Abad A, Piñero DP. (2017). New perspectives on the detection and progression of
keratoconus. J Cataract Refract Surg.

[2] Mas Tur V, MacGregor C, Jayaswal R, O’Brart D, Maycock N. (2017). A review of keratoconus: Diagnosis, pathophysiology, and
genetics. Surv Ophthalmol.

[3] Fan Gaskin JC, Patel DV, McGhee CN. (2014). Acute corneal hydrops in keratoconus - new
perspectives. Am J Ophthalmol.

[4] Meyer JJ, Gokul A, Crawford AZ, McGhee CN. (2016). Penetrating keratoplasty for keratoconus with and without
resolved corneal hydrops: long-term results. Am J Ophthalmol.

[5] Basu S, Reddy JC, Vaddavalli PK, Vemuganti GK, Sangwan VS. (2012). Long-term outcomes of penetrating keratoplasty for keratoconus
with resolved corneal hydrops. Cornea.

[6] Feder RS, Wilhelmus KR, Vold SD, O’Grady RB. (1998). Intrastromal clefts in keratoconus patients with
hydrops. Am J Ophthalmol.

[7] Rajaraman R, Singh S, Raghavan A, Karkhanis A. (2009). Efficacy and safety of intracameral perfluoropropane (C3F8)
tamponade and compression sutures for the management of acute corneal
hydrops. Cornea.

[8] Yahia Chérif H, Gueudry J, Afriat M, Delcampe A, Attal P, Gross H (2015). Efficacy and safety of pre-Descemet’s membrane sutures for the
management of acute corneal hydrops in keratoconus. Br J Ophthalmol.

[9] Subudhi P, Khan Z, Subudhi BN, Sitaram S. (2017). To show the efficacy of compressive sutures alone in the
management of acute hydrops in a keratoconus patient. BMJ Case Rep.

[10] Gokul A, Krishnan T, Emanuel PO, Saunders M, McGhee CN. (2015). Persisting extreme acute corneal hydrops with a giant
intrastromal cleft secondary to keratoconus. Clin Exp Optom.

[11] Pena FV, Pena AS, Araújo PG. (2003). Estudo Retrospectivo e compar ativo de quarenta e três
olhos com hidropisia aguda em quinhentos e sessenta e sete casos de
ceratocone. Arq Bras Oftalmol.

[12] Palioura S, Chodosh J, Pineda R. (2013). A novel approach to the management of a progressive Descemet
membrane tear in a patient with keratoglobus and acute
hydrops. Cornea.

[13] Tu EY. (2017). Descemet Membrane Endothelial Keratoplasty Patch for Persistent
Corneal Hydrops. Cornea.

